# Decentralization of Acid Fast Bacilli(AFB) External Quality Assurance Using Blind Rechecking for Sputum Smear Microscopy in Ethiopia

**DOI:** 10.1371/journal.pone.0151366

**Published:** 2016-03-18

**Authors:** Muluken Melese, Degu Jerene, Genetu Alem, Jemal Seid, Feleke Belachew, Yewulsew Kassie, Dereje Habte, Solomon Negash, Gonfa Ayana, Belaineh Girma, Yared K. Haile, Nebiyu Hiruy, Pedro G. Suarez

**Affiliations:** 1 Management Sciences for Health, Help Ethiopia Address the Low Performance of Tuberculosis (HEAL TB) Project, Addis Ababa, Ethiopia; 2 Amhara Regional Health Bureau, Bahir Dar, Ethiopia; 3 Oromia Regional Reference Laboratory, Addis Ababa, Ethiopia; 4 Ethiopian Public Health Institute, Addis Ababa, Ethiopia; 5 United States Agency for International Development (USAID), Addis Ababa, Ethiopia; 6 Management Sciences for Health, Center for Health Services, Arlington, Virginia, United States of America; Hebrew University, ISRAEL

## Abstract

**Introduction:**

Ethiopia achieved a rapid expansion of TB microscopic centers for acid fast bacilli (AFB). However, external quality assurance (EQA) services were, until recently, limited to few regional and sub-regional laboratories. In this paper, we describe the decentralization experience and the result of EQA using random blinded rechecking.

**Materials and Methods:**

The routine EQA quarterly report was compiled and analyzed. A positive result by the microscopic center while the EQA center reported negative result is categorized as false positive (FP). A negative result by the microscopic center while the EQA center reported positive is considered false negative (FN). The reading of EQA centers was considered a gold standard to compute the sensitivity, specificity, positive predictive (PPV) and negative predictive values (NPV) of the readings of microscopic centers.

**Results:**

We decentralized sputum smear AFB EQA from 4 Regional Laboratories (RRLs) to 82 EQA centers and enrolled 956 health facilities in EQA schemes. Enrollment of HFs in EQA was gradual because it required training and mentoring laboratory professionals, institutionalizing internal QA measures, equipping all HFs to perform diagnosis, and establishing more EQA centers. From 2012 to 2014 (Phase I), the FP rate declined from 0.6% to 0.2% and FN fell from as high as 7.6% to 1.6% in supported health facilities (HFs). In HFs that joined in Phase II, FN rates ranged from 5.6 to 7.3%. The proportion of HFs without errors has increased from 77.9% to 90.5% in Phase I HFs and from 82.9% to 86.9% in Phase II HFs. Overall sensitivity and specificity were 95.0% and 99.7%, respectively. PPV and NPV were 93.3% and 99.7%, respectively.

**Conclusion:**

Decentralizing blinded rechecking of sputum smear microscopy is feasible in low-income settings. While a comprehensive laboratory improvement strategy enhanced the quality of microscopy, laboratory professionals’ capacity in slide reading and smear quality requires continued support.

## Introduction

Direct sputum microscopy for acid-fast bacilli (AFB) using light microscopy is the most widely used tuberculosis (TB) diagnostic and monitoring tool worldwide [1–4]. Quality-assured TB microscopy is one of the key elements of DOTS in the STOP TB strategy of the World Health Organization (WHO) [4]. It is simple and cost effective and does not require sophisticated training or setup [4–6]. But it does require a very good system of quality assurance [6–7].

Quality assurance consists of quality control (QC), external quality assurance (EQA), and quality improvement (QI). To yield reliable, reproducible results, all three components should be implemented across the laboratory network [8]. Reliable AFB microscopic results such as smear positivity rates also help planners to understand the progress of TB control measures [9–11]. Implementation of EQA for microscopy helps to improve the quality of diagnosis of TB and measure the cure rates of TB patients on treatment. EQA is needed to ensure that smears are performed and interpreted correctly and that all microscopy centers perform at an acceptable level [5,11,12].

Despite rapid expansion of TB microscopic centers in Ethiopia for Ziehl-Neelsen (ZN), EQA services were, until recently, limited to a few regional and sub-regional laboratories. To fill this gap, the Ethiopian Public Health Institute (EPHI) introduced a decentralized EQA system using randomized blinded rechecking (RBRC) [7]. RBRC involves the collection of smears from the microscopy center laboratory for blinded re-reading at a regional reference laboratory (RRL) or other designated EQA center, with feedback to the microscopy center [7]. WHO has recommended this approach to evaluate the performance of AFB microscopy centers [13].

RBRC has been used successfully in many pilot and research projects [9,13–16]. In India, for example, RBRC has been used to measure the performance of laboratories and assess errors [13,16]. In other settings, it has been used for QI of diagnosis and monitoring of treatment response [17,18] and for QA where culture or fluorescent microscopies cannot be routinely used [19]. In Ethiopia, we supported the implementation of a decentralized EQA system for ZN microscopy over 1,600 health facilities (HFs) in two large regions. This paper presents the process of decentralization, its outcomes, and the factors that contributed to successful establishment of RBRC services.

## Materials and Methods

### Setting

**Fig 1:**
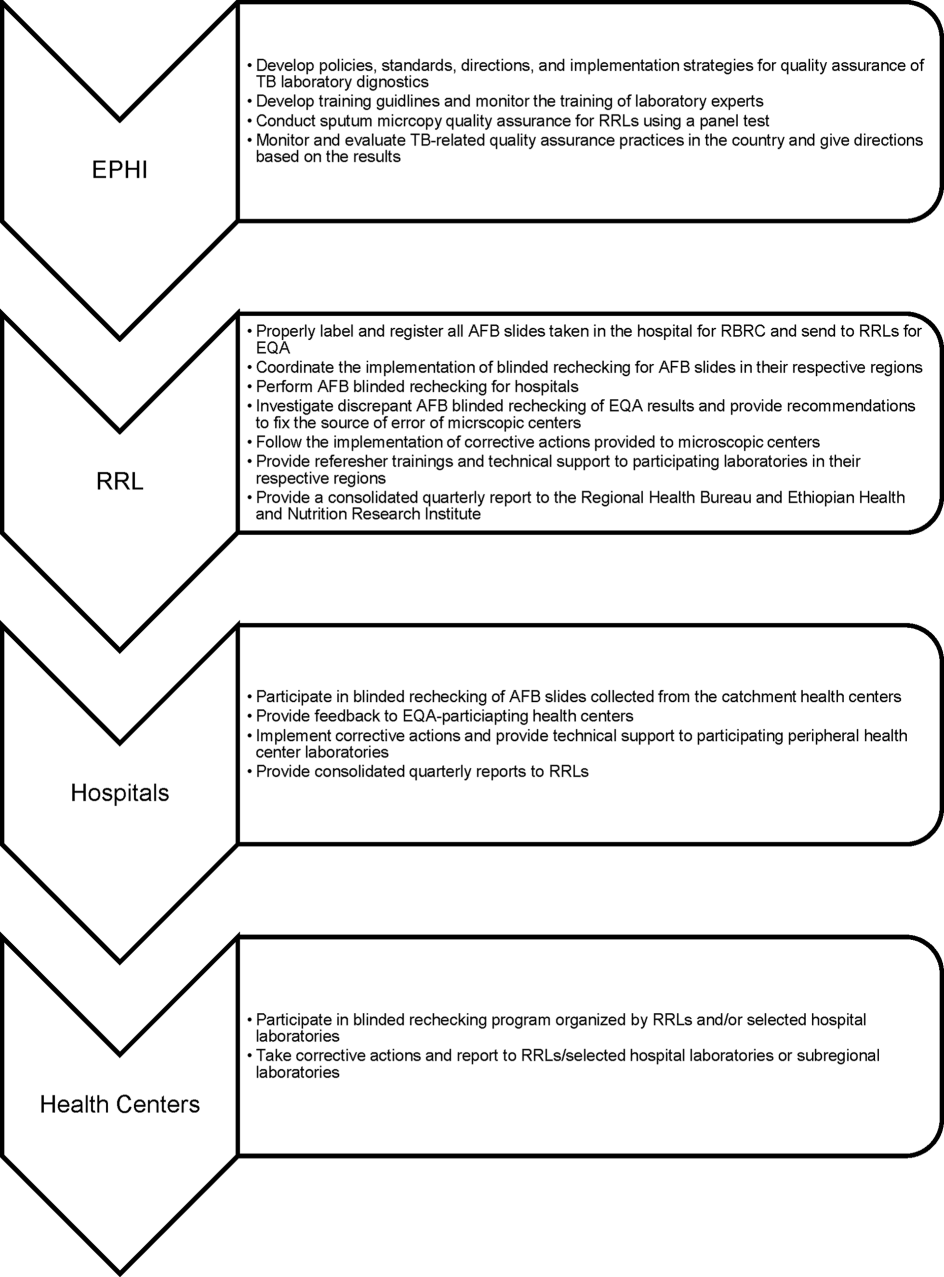
Model for decentalized AFB random blinded rechecking in Ethiopia.

In Ethiopia, which is one of the 22 high-TB-burden countries,^5^ the Federal Ministry of Health (FMOH) provides guidance for implementation of the national TB program, while the EPHI is responsible for all laboratory-related standardization and quality issues. In 2008, EPHI designed an EQA system for sputum smear Z-NAFB microscopy [7]. The system was organized so that EPHI conducts panel testing for RRLs and the RRLs conduct RBRC of sputum smear slides for hospitals. Selected hospitals with good EQA performance (≥ 95% concordance for 2–3 quarters) conduct EQA for health centers in their catchment areas (Fig 1).

### Operationalization of decentralized EQA

The Amhara and Oromia Regional Health Bureaus (RHBs) receive support from the US Agency for International Development (USAID) through the Help Ethiopia Address the Low Performance of TB (HEAL TB) project managed by Management Sciences for Health. The RHBs operationalized the decentralized EQA model through a process that involved several stakeholders.

When the project began in 2011, QA measures for AFB were weak and in most cases HFs had no QA mechanism. Following a baseline assessment, HEAL TB supported the RHBs to design a decentralized EQA system. The support included training laboratory personnel, providing standard registers, supplying microscopes and reagents, and providing quarterly supportive supervision and on-site technical support to every HF. During supervision visits, laboratory experts checked for complete registration and proper storage of the sputum smear slides and sequential labeling. The senior laboratory expert assisted in establishing internal QA measures during the site visits, including weekly checking of reagent quality with five known negative and positive slides. When errors were identified, the laboratory experts explored the cause of the error and took corrective measures with the HF laboratory personnel. After two or three quarters of follow-up and on-site support, the HFs were ready to join the country’s RBRC scheme. To assure their quality, reagents were prepared at national or regional level and distributed to all health facilities.

### Training of district TB focal persons as supervisors and slide randomization

Once the HFs were prepared for EQA participation, trained *woreda* (district) TB focal persons took the lead in supervising them. The woreda TB focal persons were trained in supervisory skills for laboratories, including checking for proper registration, labeling, and storage of slides. Every quarter the woreda TB focal persons supervised each HF in their catchment area and randomized slides for blinded rechecking following the Lot Quality Assurance Sampling guidelines for AFB slides. The nationally agreed-on sample size is based on 80% sensitivity, 100% specificity, and accepting number *d* = 0 (Table 1) [20]. The TB focal persons then delivered the collected slides to the RRLs or EQA hospitals to conduct the EQA. The EQA readers were laboratory experts from the RRLs or hospitals, who were different from those who randomized the slides.

**Table 1:** Sample size based on annual slide volume and slide positivity rate.

Number of negative slides in the microscopic center in a year	Annual sample size of both positive and negative slides for EQA(quarterly sample sizes appear in parenthesis)				
	2.5–4.9	5.0–7.49	7.5–9.9	10–14.9	15 and above
301–500	243(62)	154(40)	114(30)	89(23)	62(16)
501–1,000	318(81)	180(45)	128(33)	96(25)	66(17)
>1,000	456(114)	216(54)	144(37)	104(27)	69(18)

### Blind re-checking procedures

**Fig 2:**
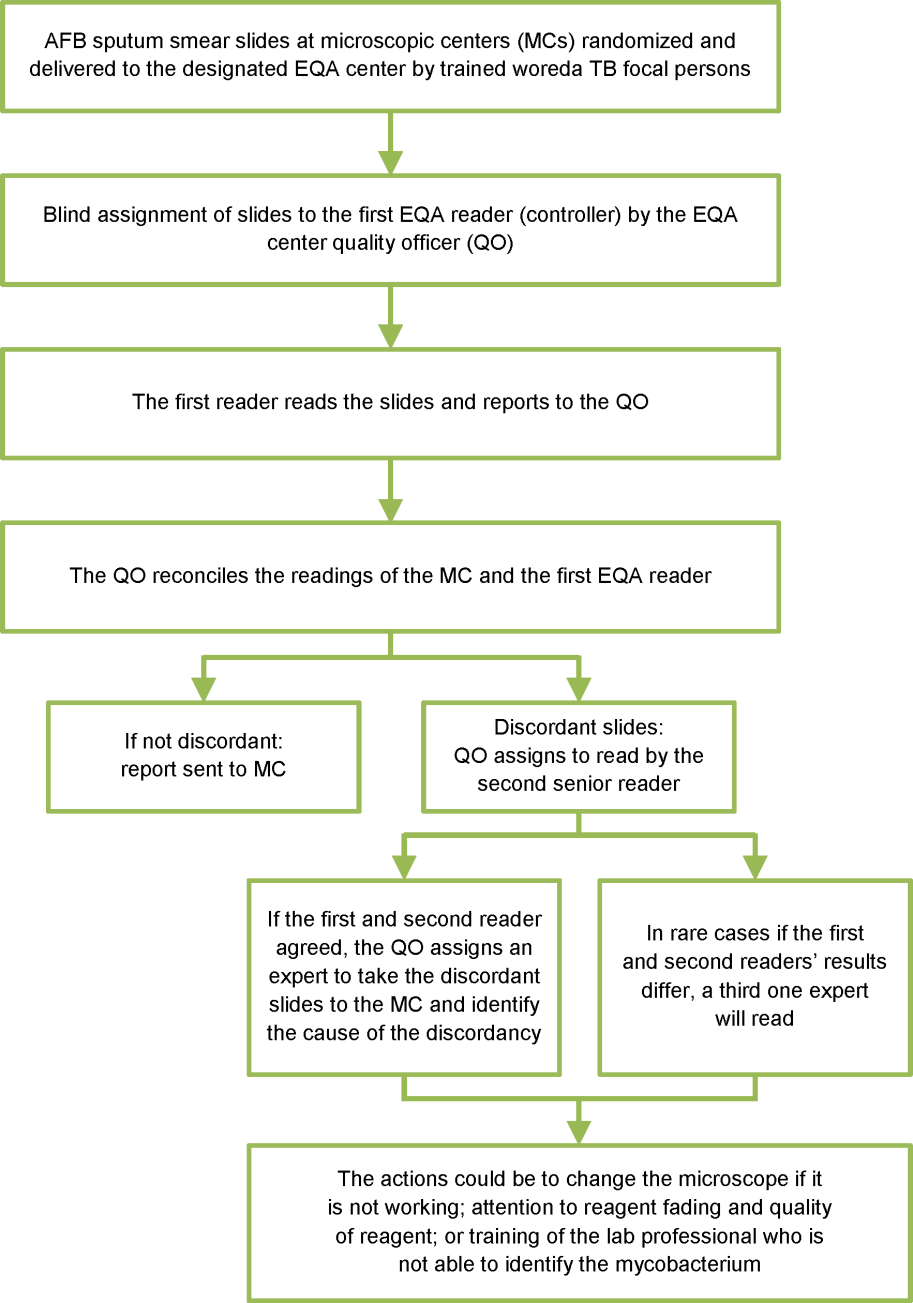
Flow chart of procedures for blinded random rechecking.

The experts involved in EQA reading have demonstrated a 95% concordance rate for at least two quarters. EPHI assesses the RRLs’ EQA through panel testing, since EPHI does not routinely collect slides for patient care. RRLs check hospitals’ EQA quarterly, and designated hospitals check the EQA of health centers (Fig 1). The quality officer of the EQA center assigns slides to a reader (controller). After the first reader completes the reading, the result is submitted to the quality officer to reconcile with the initial reading of the microscopic center. The quality officer assigns all discrepant slides from the first reader to a second reader (senior expert). If the result of the second reader agrees with that of the first, it becomes the final result. If the readings are still discordant, a third expert reads the slide, and any two concordant expert readings become the EQA result. The RRLs or hospital controllers travel to health facilities with discordant slides to identify the cause of the discordance (e.g., poorly functioning microscope, reader capacity, quality of reagent, or fading). The final result is recorded after agreement with the HF laboratory professionals is reached. (Fig 2). If the final result is different from the original report of the microscopic center, it is communicated to the treating clinicians for decision making Each EQA center covers 11–15 HFs (Table 2), and controllers conduct EQA mostly in their spare time and are paid overtime. EQA reading takes one month, and on-site evaluations of HFs with discordant slides, takes another two months.

**Table 2:** AFB microscopy EQA coverage for all HEAL TB-supported zones, April 2012-June 2014.

Indicator	April-June 2012	July-Sept 2012	Oct-Dec 2012	Jan-Mar 2013	April-June 2013	July-Sept 2013	Oct-Dec 2013	Jan-Mar 2014	April-June 2014
Total number of HFs participating in EQA RBRC	353	413	533	607	583	773	872	956	895
Total number of slides collected for EQA	13,809	16,275	22,421	27,477	22,805	30,681	37,086	41,323	36,955
Total number of EQA centers	22	38	39	40	40	56	74	80	82
Ratio HF to EQA center	16	11	14	15	15	14	12	12	11

### Data management and analysis

The reading results of peripheral laboratories were entered in Excel, the EQA results tabulated, and the analysis done based on internationally accepted definitions. The false-positive and false-negative errors were calculated using standard definitions [21]. A positive result by the microscopic center while the EQA center reported negative result is categorized false positive (FP). Similarly, if the microscopic center indicates a negative result while the EQA center reported positive, it is considered false negative (FN). Sensitivity, specificity, and positive and negative predictive values of the readings were then calculated using the EQA center controller’s final result as a gold standard, per the international guideline [20].

## Ethical Considerations

The Ethiopian Public Health Institute (EPHI) has released an AFM microscopy EQA guideline to be implemented in all microscopy centers and using a Lots Quality Assurance, sputum smear slides collected through to the routine clinical practice are randomized for RBRC. The data for this paper is acquired through this routine lab quality monitoring system, but not collected from patients directly for research purpose. As per the guiding, the sputum smear slides randomized have no patient identification information and the result is reported to evaluate the lab performance, but not directly related to patient management. EPHI has given the permission to publish the experience from the nationally reported data as the practice of decentralized EQA system has much application for low-income countries.

## Results

### Baseline data

During the first phase of project implementation (July 2011-June 2013), 691 DOTS-providing HFs were supported through HEAL TB in Amhara and Oromia regions. At baseline in October 2011, 465 HFs were providing TB diagnostic services, and 104 were participating in sporadic AFB on-site quality checks but not RBRC using proper sampling. By the end of 2013, the remaining 226 non-diagnostic HFs were equipped to become diagnostic and all 629 were enrolled in RBRC. During the second phase of the project (July 2013-present), 909 more HFs were included and at the time of this analyses 335 of them were enrolled in RBRC. The rest were under mentorship to be part of the RBRC scheme.

### Trends in EQA participation

The number of diagnostic HFs participating in RBRC increased from none at baseline to 956 by the end of March 2014. Ninety-one percent of the 691 DOTS centers in Phase I HFs were able to participate in the quarterly RBRC scheme, while the remaining 9% were checked on site because of low slide volume. Of the Phase II HFs, 37% have started to participate in EQA. In June 2014, the number of EQA-participating facilities decreased because the HFs with heavy patient loads shifted from Ziehl-Neelsen microscopy to iLED (light-emitting diode) fluorescence microscopy. Enrollment of HFs in EQA was gradual because it required training and mentoring laboratory professionals, institutionalizing internal QA measures, equipping all HFs to perform diagnosis, and establishing more EQA centers (Table 2).

**Fig 3:**
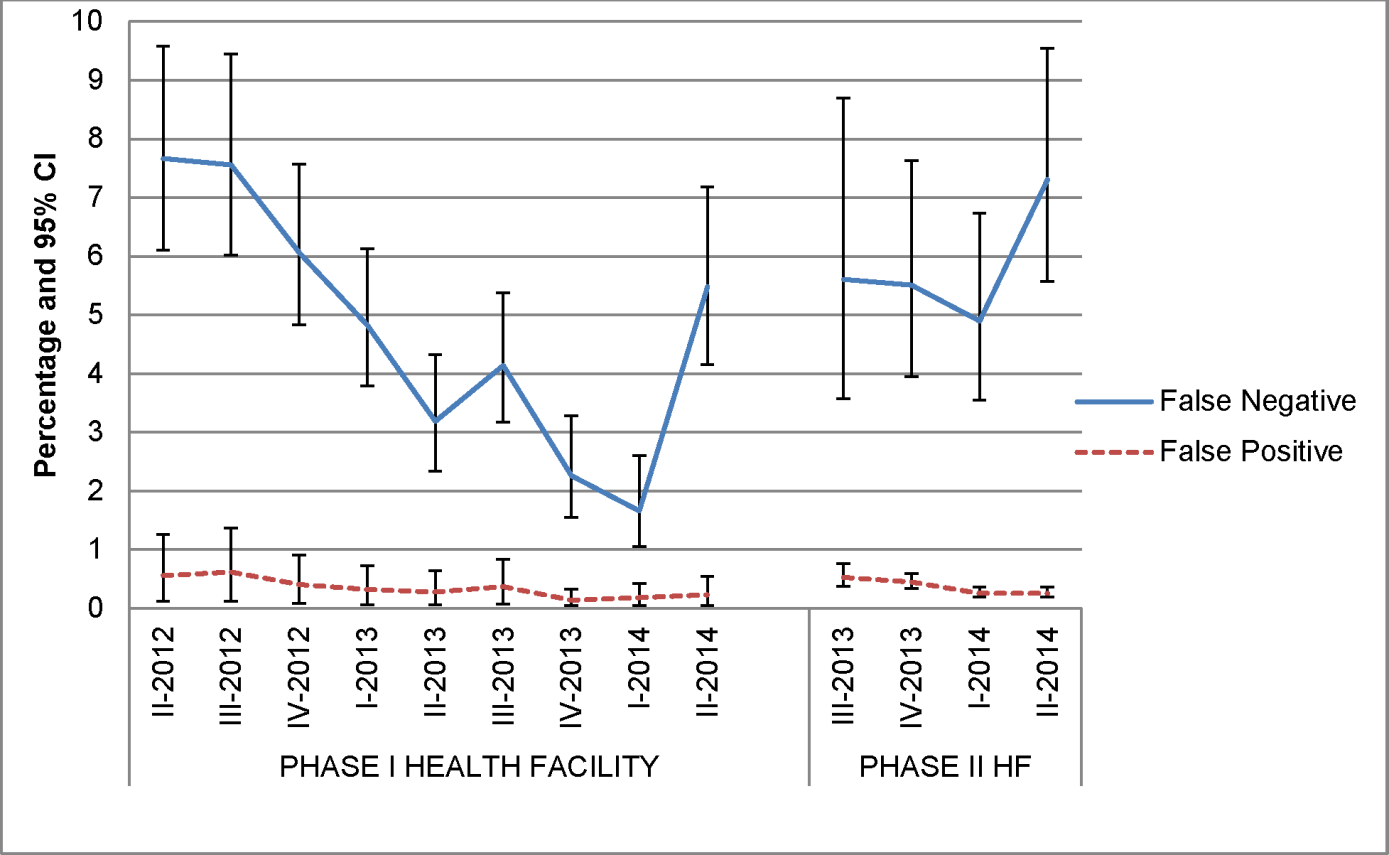
Health facilities’ reported false-negative and false-positive error rates per quarter, April 2012-June 2014.

**Fig 4:**
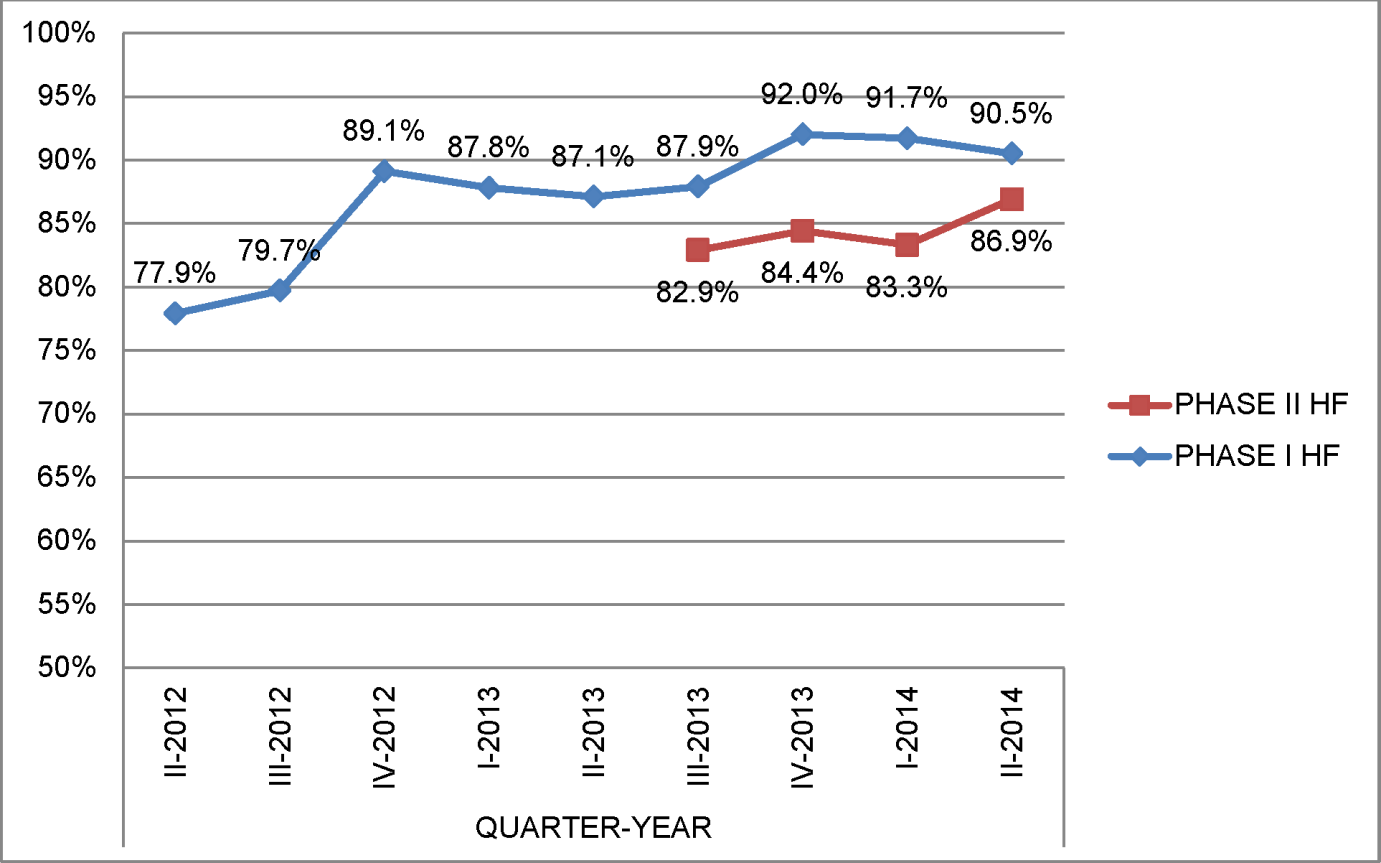
Percentage of health facilities without any error per quarter, April 2012-June 2014.

The EQA centers grew from 4 at baseline to 82 by the end of June 2014 (Table 2). Between April 2012 and June 2014, the EQA centers had read 248,832 slides. In Phase I HFs the false-positive rate declined from 0.6% (95% CI, 0.4–0.7) to 0.2% (95% CI, 0.2–0.3) and false negatives had a steady decline from 7.6% (95% CI, 6.1–9.6) to 1.6% (95% CI, 1.0–2.6) over two years, with a slight increase in the last quarter in Phase I HFs (Table 3, Fig 3). The denominator used to calculate the false negative is positive readings and for that false positives is negative readings. The proportion of HFs with no errors at this increase in Phase I reached 90.5% as opposed to 77.9% at the beginning of the project (Fig 4). In Phase II HFs the false-negative rate ranged from 5.6% to 7.3% while false positives ranged from 0.5% to 0.3% (Table 3, Fig 3).

**Table 3:** False-negative and false-positive rates per quarter, April 2012-June 2014.

Quarter-Year	Phase I: Implementation Zones			
	Number of slides collected from microscopic centers		Error rates reported by the EQA centers	
	Negative results by the laboratory	Positive rate by the laboratory	%[95%CI] false-negative slides	%[95%CI] false-positive slides
II- 2012	12,894	915	7.6 [6.09, 9.56]	0.6 [0.44, 0.70]
III- 2012	15,373	902	7.5 [5.78, 9.21]	0.6 [0.51, 0.76]
IV- 2012	21,248	1,173	6.0 [4.74, 7.48]	0.4 [0.33, 0.51]
I- 2013	26,171	1,306	4.8 [(3.71, 6.05]	0.3 [0.26, 0.40]
II- 2013	21,563	1,242	3.1 [2.29, 4.27]	0.3 [0.22, 0.36]
III- 2013	23,444	1,268	4.0 [3.06, 5.26]	0.4 [0.30, 0.46]
IV- 2013	24,420	1,203	2.2 [1.53, 3.26]	0.1 [0.09, 0.19]
I- 2014	25,158	1,114	1.6 [(1.01, 2.56]	0.2 [0.14, 0.24]
II- 2014	21,046	894	5.2 [4.16, 7.18]	0.2 [0.18, 0.31]
Phase II: Implementation Zones				
Quarter-Year	Negative results by the laboratory	Positive rate by the laboratory	%[95%CI] false-negative slides	%[95%CI] false-positive slides
III- 2013	5,636	333	5.6 [3.39, 8.43]	0.5 [0.37, 0.76]
IV- 2013	10,848	615	5.4 [3.82, 7.46]	0.4 [0.34, 0.59]
I- 2014	14,335	716	4.9 [3.52, 6.74]	0.3 [0.19, 0.36]
II- 2014	14,356	659	7.3 [5.65, 9.71]	0.3 [0.19, 0.36]

By the end of the study, overall sensitivity and specificity for the Phase I HFs were 95% and 99.7%, respectively, and the positive predictive value (PPV) and negative predictive value (NPV) were 93.7% and 99.7% respectively. In Phase II HFs, sensitivity and specificity were 94.1% and 99.6% respectively. The PPV and NPV were 93.3% and 99.7% respectively.

In Phase I HFs, the average quality of staining at baseline was 71.1% and by June 30, 2014, it reached 81.4%. In Phase II HFs, it increased from 61.7% at baseline to 72.7% by June 2014. Smear thickness also improved, from 62.1% to 69.8% in Phase I HFs, but in Phase II HFs it improved from 59.3% to 71% and then decreased to 57.0%. In Phase I HFs cleanliness of the slides improved from 72.6% to 86.3%, but in Phase II HFs cleanliness improved from 80.7% to 88.4% and then declined to 81.6% in June 2014 (Table 4).

**Table 4:** Sputum smear quality assessment by the EQA centers, April 2012-June 2014.

**Phase I Implementation Zones**						
**Quarter**	**Number of HFs enrolled in EQA**	**Total number of sampled slides**	**Good-quality staining (%)**	**Smear thickness (%)**	**Cleanliness of slides (%)**	**Evenness of smearing (%)**
II- 2012	353	13,809	71.1	62.1	72.6	53.0
III- 2012	413	16,275	69.8	62.5	82.4	59.1
IV- 2012	533	22,421	72.9	67.1	83.7	63.7
I- 2013	607	27,477	74.9	71.1	84.8	66.1
II- 2013	583	22,805	75.4	70.3	87.1	67.5
III- 2013	626	24,712	75.1	67.7	85.2	67.2
IV- 2013	603	25,623	77.2	71.8	88.3	69.7
I- 2014	614	26,272	78.2	70.9	85.4	68.8
II- 2014	560	21,940	81.4	69.8	86.3	66.6
*Total*		*201*,*334*				
**Phase II Implementation Zones**						
**Quarter**	**Number of HFs enrolled in EQA**	**Total number of sampled slides**	**Good-quality staining (%)**	**Smear thickness (%)**	**Cleanliness of slides (%)**	**Evenness of smearing (%)**
III- 2013	147	5,969	61.7	59.3	80.7	54.7
IV- 2013	269	11,463	63.9	59.7	79.7	51.7
I- 2014	342	15,151	77.0	71.0	88.4	65
II- 2014	335	15,015	72.7	57.0	81.6	55.0
*Total*		*47*,*498*				

## Discussions

This study demonstrates that decentralizing AFB EQA services to hospitals is a feasible, low-cost approach for countries like Ethiopia that have few higher-level laboratories [8,13,22,23]. With the decentralized approach, nearly 1,000 HFs participated in RBRC. It takes approximately 6–9 months to prepare the HFs for RBRC, but in Phase II, 335 HFs enrolled in EQA, which was faster than expected because of the experience gained during Phase I implementation.

The rapid capacity building of HFs in sputum smear microscopy, coupled with on-site supervision, helped decrease the numbers of false-positive and false-negative slides. Other countries have reported improvements using similar mechanisms [8,18] Our experience is that the proportion of HFs with no errors improved from quarter to quarter but the error rate fluctuated because of HFs enrolled in EQA for the first time or new, less-trained laboratory professionals assigned to the HFs. The HFs began EQA in different phases, but in three quarters the false-negative rate declined significantly (Table 3). In 507 Phase I HFs with EQA results, for example, in April-June 2014, only 48 contributed to the reported errors. False-positive errors were low from the beginning, and there was statistically significant improvement in both phases.

Another possible reason for errors was smear quality, although there were improvements from quarter to quarter because of comprehensive capacity building (Table 4). We addressed challenges by providing refresher training at sites with poor performance and at laboratories with new personnel. In addition, the EQA centers served as mentors and trainers for new laboratory professionals and underperforming laboratories. Every week the HF also checks reagent quality with known negative and positive slides. RRL staff also visit and identify the causes of errors with the HFs’ laboratory experts. If fading is suspected, they re-stain the slides and read them on-site with the same microscope used for diagnosis by the HF.

The overall sensitivity of 95.0% and specificity of 99.7% in our health facilities are high, per international standards [20], and the national recommendation about the sample size for RBRC for Ethiopia might need revision. The revised international recommendation for EQA of AFB smear microscopy is to use a 75–80% sensitivity rate to calculate sample sizes for blinded rechecking [7,20]. Ethiopia has already adopted this recommendation, so the sample size for blinded rechecking was calculated based on a sensitivity of 80% [7]. Future samples will be large, if Ethiopia plans to revise the sampling based on the improved EQA results. EQA centers may be overloaded with large numbers of slides.

The decentralized approach is cost effective because the EQA readers and district TB focal persons who randomize slides for EQA are all government workers paid according to government rates. EQA readers are paid US$5 per 20 slides in Oromia Region and US$2 in Amhara. The per diem and transport for the district focal person is US$7.50 per HF. These costs are manageable for the government, which will help to sustain the system. The experience in scaling-up of EQA, the progressive improvement in quality and the cost-effectiveness of the approach heralds that such system can easily be easily replicated in similar settings.

There are some limitations of the study. The false-positive and false-negative rates at EQA center level were not reported using the scanty, 1+, 2+, and 3+ categories, but for four quarters in the initial period the regions were reporting summary data, so we could not compute error rates by category. Therefore the analysis is limited to false positives and false negatives rather than detailed classifications. The data did not capture whether the AFB slides included in the EQA were collected for diagnostic purpose or TB treatment follow-up. As a result, we were not able to compare the EQA in the two groups independently. However, the regularity of data collection and the huge number of HFs covered represent strengths of this study.

## Conclusion

A decentralized EQA scheme was feasible in a large number of HFs in Ethiopia. Involving hospitals has contributed to rapid scale-up of the EQA scheme to thousands of HFs every quarter. AFB quality has improved gradually and error rates have declined in many HFs. The model is scalable and sustainable because it was designed and built within the Ethiopian health care system. Close on-site mentoring of DOTS centers and of HFs with errors are critical for the success of this approach. Pre-placement trainings for newly assigned laboratory personnel should be implemented routinely to prevent the high error rates reported from sites with new staff. Smear quality improvement is a priority to further reduce errors. The overall impact of the decentralized EQA scheme on improving the quality of TB care should be evaluated. A clear sputum sample transport to the expanding GeneXpert and culture centers should be established to improve the diagnosis of TB those cannot be by microscopy.
